# Calcium Beta‐Hydroxy‐Beta‐Methylbutyrate‐Enriched Nutritional Supplementation During Dietary Weight Loss in Adults With Obesity: A Randomized Controlled Trial

**DOI:** 10.1002/jcsm.70343

**Published:** 2026-07-13

**Authors:** Jiaojiao Jiang, Yuxiang Liang, Renjie Wang, Wenhua Jiang, Xiaofan Jing, Ming Yang

**Affiliations:** ^1^ Rehabilitation Center West China Hospital, Sichuan University Chengdu China; ^2^ Key Laboratory of Rehabilitation Medicine in Sichuan Province Chengdu China; ^3^ West China Fourth Hospital, Sichuan University Chengdu China; ^4^ Department of Clinical Nutrition West China Hospital, Sichuan University Chengdu China; ^5^ National Clinical Research Center for Geriatrics West China Hospital, Sichuan University Chengdu China; ^6^ Center of Gerontology and Geriatrics West China Hospital, Sichuan University Chengdu China; ^7^ Institute of Respiratory Health and Multimorbidity West China Hospital, Sichuan University Chengdu China

**Keywords:** beta‐hydroxy‐beta‐methylbutyrate, body composition, caloric restriction, nutritional supplementation, obesity, skeletal muscle mass, visceral fat, weight loss

## Abstract

**Background:**

Dietary and pharmacologic weight loss can reduce skeletal muscle. Whether calcium beta‐hydroxy‐beta‐methylbutyrate (CaHMB)‐enriched supplementation preserves muscle while promoting fat loss in adults with obesity remains uncertain. We evaluated a CaHMB‐enriched nutritional supplement during energy restriction.

**Methods:**

In this randomized, double‐blind, controlled trial, 102 Chinese adults with obesity (body mass index [BMI] ≥ 28 kg/m^2^) were assigned 1:1 to a CaHMB‐enriched nutritional supplement (65 g/day powder containing 3.0 g/day CaHMB and 24 g/day protein) or an energy‐matched maltodextrin control for 12 weeks, alongside standardized dietary energy restriction. The primary outcome was change in whole‐body skeletal muscle mass (SMM) by multifrequency bioelectrical impedance analysis. Secondary outcomes included visceral fat area (VFA), body fat mass (BFM), basal metabolic rate (BMR), physical function and metabolic biomarkers. ITT analyses adjusted for baseline values, age, sex, dietary intake and physical activity changes.

**Results:**

Participants had a mean age of 36.8 ± 5.2 years, a mean BMI of 32.2 ± 3.3 kg/m^2^ and 37.3% (38/102) were female. The CaHMB group maintained SMM (median +0.7 kg; IQR = −0.2 to 2.0), whereas the control group lost SMM (median = −0.6 kg; IQR = −1.9 to −0.1; between‐group difference 1.3 kg; 95% CI = 0.5 to 1.9; *p* < 0.001). More CaHMB participants gained ≥ 0.5 kg SMM (52.9% vs. 11.8%; OR = 8.44; 95% CI = 3.23 to 25.22; *p* < 0.001). CaHMB also produced greater reductions in VFA (adjusted mean difference [AMD] = −14.9 cm^2^; 95% CI = −25.1 to −4.5; *p* = 0.004) and BFM (AMD = −4.9 kg; 95% CI = −6.8 to −3.1; *p* < 0.001), preserved BMR (difference 62.0 kcal/day; 95% CI = 35.0 to 89.0; *p* < 0.001) and reduced fasting glucose more than control (difference −0.4 mmol/L; 95% CI = −0.8 to −0.1; *p* = 0.009). No adverse events were reported.

**Conclusions:**

During dietary weight loss, CaHMB‐enriched supplementation preserved SMM and produced greater visceral and total fat reductions. Because the intervention also contained protein, longer factorial trials are needed to isolate CaHMB‐specific effects.

**Trial Registration:**

ClinicalTrials.gov identifier: NCT04953936

**Abbreviations**
ALT: alanine aminotransferase
ANCOVA: analysis of covariance
ASMM: appendicular skeletal muscle mass
AST: aspartate aminotransferase
BFM: body fat mass
BIA: bioelectrical impedance analysis
BMI: body mass index
BMR: basal metabolic rate
CaHMB: calcium beta‐hydroxy‐beta‐methylbutyrate
CI: confidence interval
CTCAE: Common Terminology Criteria for Adverse Events
DXA: dual‐energy x‐ray absorptiometry
eGFR: estimated glomerular filtration rate
FFQ: food frequency questionnaire
FTSST: five times sit‐to‐stand test
GGT: gamma‐glutamyl transferase
GLP‐1: glucagon‐like peptide‐1
HDL‐C: high‐density lipoprotein cholesterol
HGS: handgrip strength
HOMA‐IR: homeostatic model assessment for insulin resistance
IPAQ‐SF: International Physical Activity Questionnaire Short Form
IQR: interquartile range
ITT: intention‐to‐treat
LDL‐C: low‐density lipoprotein cholesterol
PP: per‐protocol
SD: standard deviation
SMM: skeletal muscle mass
VFA: visceral fat area

## 1 Introduction

Obesity is a complex, multifactorial chronic disease affecting nearly 1 billion people globally, serving as a major driver for cardiovascular diseases, type 2 diabetes and malignancy [1, 2]. While lifestyle interventions remain the foundation of management, the landscape of obesity treatment has been revolutionized by the advent of potent pharmacotherapies. A recent network meta‐analysis demonstrated that glucagon‐like peptide‐1 (GLP‐1) receptor agonists, such as semaglutide, and dual GIP/GLP‐1 agonists like tirzepatide, can achieve weight losses of 15%–20%, rivalling the efficacy of bariatric surgery [3, 4]. However, this therapeutic breakthrough has unveiled a critical physiological challenge: the substantial loss of lean body mass. Emerging evidence indicates that up to 40% of the weight lost with these potent agents is attributable to skeletal muscle, raising concerns about the long‐term risk of sarcopenia and functional decline, particularly in older adults [5, 6].

This ‘collateral damage’ to skeletal muscle disrupts the fundamental goal of obesity management, which, as highlighted by the latest Lancet Commission on Obesity, should prioritize the improvement of health outcomes rather than weight loss alone [7, 8]. This issue is particularly salient in the Western Pacific region, where populations often exhibit a ‘thin‐fat’ phenotype—higher body fat percentages and greater visceral adiposity at lower BMI thresholds compared to Caucasians [9]. Consequently, individuals in this region are disproportionately susceptible to sarcopenic obesity, a high‐risk phenotype characterized by the coexistence of excess adiposity and muscle insufficiency [10]. Therefore, identifying strategies to uncouple weight loss from muscle loss—achieving ‘high‐quality weight loss’—is an urgent unmet medical need, especially for Asian populations.

Calcium beta‐hydroxy‐beta‐methylbutyrate (CaHMB), a bioactive metabolite of leucine, acts as a potent regulator of muscle protein turnover by increasing muscle protein synthesis through stimulation of the mTORC1 pathway and decreasing muscle protein degradation by inhibiting the ubiquitin‐proteasome proteolytic system [11]. While its efficacy in sarcopenia and sports nutrition is established, its role in obesity management remains under‐explored. Current guidelines for obesity emphasize the heterogeneity of metabolic phenotypes and the need for precision approaches [12, 13]. In this context, CaHMB could offer a targeted nutritional strategy to preserve muscle mass during energy restriction. To test this, we conducted a randomized, double‐blind, placebo‐controlled trial to evaluate the efficacy of CaHMB‐enriched nutritional supplements on skeletal muscle preservation and body composition optimization in adults with obesity.

## 2 Methods

### 2.1 Study Design and Participants

This single‐centre, randomized, double‐blind, controlled, parallel‐group trial was conducted at West China Hospital, Sichuan University, Chengdu, China. The recruitment of participants took place from March to April 2022, with a subsequent 12‐week intervention period for each participant. The study protocol adhered to the ethical principles of the Declaration of Helsinki and was approved by the Biomedical Ethics Committee of West China Hospital (Approval No. 2021‐771). The detailed study protocol has been published previously [14]. The trial was registered at ClinicalTrials.gov (NCT04953936) prior to participant enrolment. Written informed consent was obtained from all participants. This study is reported in accordance with the Consolidated Standards of Reporting Trials (CONSORT) reporting guideline.

Eligible participants were adults aged 30–50 years with obesity, defined as a body mass index (BMI) ≥ 28 kg/m^2^, who intended to lose weight via caloric restriction. Inclusion criteria further required participants to be capable of independent ambulation and feeding, to have maintained a stable body weight (change < 5%) over the preceding 6 months and to exhibit a sedentary lifestyle as defined by the Sedentary Behaviour Research Network [15]. Exclusion criteria included a history of intolerance or allergy to the supplement components (e.g., soy, dairy and lactose); presence of implanted medical devices; secondary obesity induced by endocrine disorders or medications; clinically visible oedema; or significant trauma, surgery or fracture within the past 6 months. Participants were also ineligible if they were pregnant, lactating or planning pregnancy; had acute illnesses; or suffered from chronic systemic conditions, including uncontrolled hypertension, diabetes, cardiovascular disease, renal or hepatic insufficiency (excluding fatty liver) or malignancy. Individuals currently using medications or supplements affecting muscle mass or weight (e.g., glucocorticoids and weight‐loss drugs) and those with excessive alcohol consumption (> 20 g/day) were excluded.

### 2.2 Randomization and Masking

Participants were randomly assigned in a 1:1 ratio to either the CaHMB group or the control group using a stratified blocked randomization method. The randomization sequence was generated using R software (Version 4.0.3), with gender serving as the stratification factor to ensure a balanced sex distribution. Within each gender stratum, block sizes ranging from 2 to 8 were randomly selected. Allocation concealment was maintained using sequentially numbered, opaque, sealed envelopes, which were opened by independent research staff only at the commencement of the intervention. To ensure double‐blinding, the intervention and control supplements were provided in identical opaque packages and were similar dry‐powder preparations. Participants, outcome assessors and statistical analysts remained blinded to group allocation until trial completion and data lock.

### 2.3 Procedures

All participants underwent a standardized lifestyle intervention consisting of dietary caloric restriction and nutritional guidance. Individualized dietary instructions were provided by professional dietitians via video consultations. The daily energy intake target was calculated based on ideal body weight using the formula: (Height [cm] ‐ 105) × 25 kcal/kg/day. Adherence to the dietary prescription was monitored weekly through WeChat and telephone follow‐ups. Dietary counselling was provided weekly during the first 4 weeks and biweekly during weeks 5–12, for a total of approximately eight consultations per participant. Consultations followed a standardized protocol and were delivered by the same team of dietitians to both groups.

In addition to the lifestyle intervention, participants in the intervention group received a CaHMB‐enriched nutritional solid beverage (cheese flavour). Each daily dose consisted of 65 g of powder, providing 994.4 kJ (approximately 237 kcal) of energy, 24.0 g of protein, 9.3 g of fat, 14.6 g of carbohydrates and 9.5 g of dietary fibre. The formulation contained 3.0 g of calcium beta‐hydroxy‐beta‐methylbutyrate (CaHMB) per daily dose, together with soy isolate protein, flaxseed oil powder, whey protein, casein, L‐glutamine, medium‐chain triglycerides, wheat oligopeptides and vitamins D and E. Participants in the control group received 65 g/day of maltodextrin as an energy‐matched control without CaHMB, protein or additional micronutrients. Participants were instructed to dissolve the powder in 300 mL of warm water and consume it once daily, preferably with or immediately after breakfast, for 12 consecutive weeks. The recommended timing was not strictly enforced. Adherence to the supplement and dietary prescription was monitored through WeChat and telephone follow‐up, review of participant daily logs and returned containers when available; a group‐specific quantitative adherence percentage was not available in the locked analysis dataset.

### 2.4 Outcomes

Outcomes were assessed at baseline, week 6 and week 12. The primary outcome was the mean change in whole‐body skeletal muscle mass (SMM) from baseline to week 12. Body composition, including SMM, appendicular skeletal muscle mass (ASMM), body fat mass (BFM), visceral fat area (VFA) and phase angle (PhA), was measured using multifrequency bioelectrical impedance analysis (BIA) (InBody 770; Biospace Co., Seoul, South Korea). The InBody 770 has demonstrated high correlation (*r* > 0.9) with dual‐energy x‐ray absorptiometry (DXA) for measuring appendicular lean mass in Asian populations [16]. To minimize hydration‐related variability and ensure data quality, measurements were performed under strictly standardized conditions: Participants were required to fast for at least 8 h overnight, void their bladder immediately before assessment and rest in a seated position for 15 min prior to measurement. All assessments were conducted between 8:00 and 10:00 A.M. by the same trained technician. Participants were instructed to stand upright barefoot on the platform with standardized posture during the scan.

Secondary outcomes included changes in anthropometric indices, physical function and metabolic biomarkers. Anthropometric measurements comprised body weight, BMI, waist circumference and hip circumference. Muscle function was evaluated using handgrip strength (HGS) and the five times sit‐to‐stand test (FTSST). HGS was measured using a digital handheld dynamometer (EH101; Xiangshan Inc., Guangdong, China), with the maximum value of three trials per hand recorded. The FTSST measured the time required to complete five full stands from a seated position, serving as a proxy for lower extremity muscle strength. Physical activity levels and dietary intake were monitored using the International Physical Activity Questionnaire Short Form (IPAQ‐SF) [17] and a Food Frequency Questionnaire (FFQ), respectively, assessed at baseline and weeks 3, 6, 9 and 12. The IPAQ‐SF was utilized to adequately monitor group balance in physical activity, a key potential confounder, throughout the intervention.

Metabolic and safety profiles were assessed via fasting blood samples collected between 7:00 and 9:00 AM after an overnight fast of at least 8 h at baseline and week 12. Fasting glucose, lipid profiles, albumin, liver enzymes, renal function markers and complete blood counts were measured by the clinical laboratory of West China Hospital using standard clinical chemistry and haematology methods. Fasting insulin was measured using the hospital laboratory's standard immunoassay platform. HOMA‐IR was calculated from fasting glucose and fasting insulin according to the established formula [18]. Safety outcomes involved the monitoring of adverse events (graded according to CTCAE Version 5.0) [19] and laboratory safety parameters, including liver function enzymes (ALT, AST and GGT), renal function markers (creatinine, cystatin C and eGFR) and complete blood counts.

### 2.5 Sample Size Estimation

The sample size calculation was based on the primary outcome of change in SMM. Referencing prior studies on CaHMB supplementation (Wilkinson et al., 2013 [11]; Lin et al., 2021 [20]), an effect size of 0.93 was assumed. To detect a significant difference with 80% power at a two‐sided significance level of 0.05, and accounting for an anticipated attrition rate of 25%, a total sample size of 72 participants (36 per group) was initially determined to be sufficient. The study ultimately recruited 102 participants to ensure adequate power for subgroup analyses.

### 2.6 Statistical Analysis

All statistical analyses were conducted using R software (Version 4.3.1, R Foundation for Statistical Computing, Vienna, Austria) [21] and SPSS software (Version 26.0, IBM Corp., Armonk, NY, USA). A two‐sided *p* value of less than 0.05 was considered statistically significant. Continuous variables were first evaluated for normality using the Shapiro–Wilk test and visual inspection of histograms and Q‐Q plots. Normally distributed data were presented as mean ± standard deviation (SD), while nonnormally distributed data were expressed as median with interquartile range (IQR). Categorical variables were reported as frequencies and percentages (*n* [%]). Baseline characteristics were compared between the intervention and control groups using Student's *t*‐test or the Mann–Whitney *U* test for continuous variables, and the chi‐square test or Fisher's exact test for categorical variables, as appropriate based on data distribution and expected cell counts.

The primary efficacy analysis was performed in the intention‐to‐treat (ITT) population, which included all randomized participants (*n* = 102). Missing 12‐week outcome values in ITT analyses were addressed using multiple imputation by chained equations (MICE) to generate five imputed datasets under a missing‐at‐random assumption; estimates were pooled according to Rubin's rules [22]. The per‐protocol (PP) population (*n* = 96) comprised participants who completed the 12‐week intervention without major protocol deviations; six randomized participants were excluded from PP analyses because they withdrew or were unable to comply with the dietary protocol. Complete‐case and PP analyses were performed as sensitivity analyses. Within‐group changes from baseline to week 12 were evaluated using paired *t*‐tests or the Wilcoxon signed‐rank test.

Between‐group differences in the primary outcome (change in skeletal muscle mass) and secondary outcomes were analyzed using analysis of covariance (ANCOVA), generalized linear models or median regression according to outcome distribution. Three hierarchical models were constructed: Model 1 was unadjusted; Model 2 was adjusted for baseline values of the outcome, age and sex; and Model 3 (the primary analysis model) was further adjusted for changes in dietary energy intake, dietary protein intake and physical activity levels (IPAQ scores) during the intervention period. Treatment effects were reported as adjusted mean differences (beta) or adjusted median differences with 95% confidence intervals (CIs). For nonnormally distributed outcomes, including SMM, triglycerides, fasting insulin and HOMA‐IR when analyzed, treatment effects were estimated using median regression, with 95% CIs obtained by bootstrapping. For time‐course analyses incorporating 6‐week data, linear mixed‐effects models included fixed effects for group, categorical time and the group‐by‐time interaction, with a random intercept for participant. Standardized treatment effects in Figure 3 were calculated as Model 3 treatment coefficients divided by the SD of the change score and were not reoriented; therefore, clinical interpretation followed the natural beneficial direction of each outcome.

Exploratory and safety analyses were performed to further characterize the intervention's effects. Subgroup analyses were conducted for the primary outcome stratified by age (< 40 vs. ≥ 40 years), gender (male vs. female) and baseline BMI (< 32 vs. ≥ 32 kg/m^2^), with interaction *p* values calculated to evaluate the heterogeneity of treatment effects across these subgroups. Exploratory mediation analyses investigated potential mechanisms underlying the effect of CaHMB‐enriched supplementation on muscle mass preservation, specifically testing basal metabolic rate (BMR) and phase angle as mediators. The indirect effects were estimated using the product‐of‐coefficients method with bias‐corrected bootstrapping (1000 iterations). Responder, subgroup, mediation and secondary outcome analyses were interpreted as exploratory; no formal adjustment for multiplicity was applied. Safety parameters, including vital signs and laboratory indices, were compared between groups using Student's *t*‐test or the Mann–Whitney *U* test in the ITT population.

## 3 Results

### 3.1 Participant Flow and Recruitment

From March to April 2022, a total of 116 individuals were screened for eligibility. Of these, 102 participants met the inclusion criteria and were randomly assigned to the CaHMB group (*n* = 51) or the control group (*n* = 51). All randomized participants were included in the ITT analysis. During the 12‐week intervention period, six participants (5.9%) withdrew from the study (two in the CaHMB group and four in the control group), primarily due to personal reasons or inability to comply with the dietary protocol. Consequently, 96 participants (49 in the CaHMB group and 47 in the control group) completed the full protocol and were included in the PP analysis. The flow of participants through the trial is depicted in Figure S1.

### 3.2 Baseline Characteristics

Baseline demographic, anthropometric, metabolic and safety profiles (including liver and kidney function) were well balanced between the two groups (Table 1). The mean age of the participants was 36.8 ± 5.2 years, and 37.3% were female. There were no statistically significant differences in baseline body weight, BMI, SMM or other metabolic and safety parameters between the CaHMB and control groups (all *p* > 0.05), indicating successful randomization.

**TABLE 1:** Baseline characteristics of participants.

Characteristic	Overall (*n* = 102^a^)	Control (*n* = 51^a^)	CaHMB (*n* = 51^a^)	*p* ^b^
**Demographics**				
Age (years)	36.8 (5.2)	36.9 (5.1)	36.7 (5.4)	0.700
Sex				0.999
Male	64 (62.7%)	32 (62.7%)	32 (62.7%)	
Female	38 (37.3%)	19 (37.3%)	19 (37.3%)	
Height (cm)	167.8 (10.4)	167.4 (11.1)	168.3 (9.7)	0.771
Weight (kg)	91.4 (16.2)	90.6 (16.5)	92.2 (15.9)	0.602
BMI (kg/m^2^)	32.2 (3.3)	32.1 (3.2)	32.4 (3.5)	0.745
Education Level				0.999
Primary/middle	1 (1.0%)	0 (0.0%)	1 (2.0%)	
High school	12 (11.8%)	6 (11.8%)	6 (11.8%)	
University	89 (87.3%)	45 (88.2%)	44 (86.3%)	
**Lifestyle characteristics**				
Smoking	64 (64.0%)	31 (63.3%)	33 (64.7%)	0.881
Drinking	62 (62.6%)	31 (62.0%)	31 (63.3%)	0.896
Exercise habit				0.213
≥ 2 h/day	1 (1.0%)	1 (2.0%)	0 (0.0%)	
1–1.9 h/day	8 (7.8%)	2 (3.9%)	6 (11.8%)	
0.5–0.9 h/day	23 (22.5%)	14 (27.5%)	9 (17.6%)	
< 0.5 h/day	70 (68.6%)	34 (66.7%)	36 (70.6%)	
Energy intake (kcal/d)	2316.5 (770.1)	2267.3 (696.7)	2365.8 (841.1)	0.748
Protein intake (g/d)	81.0 (25.4)	81.2 (27.1)	80.9 (23.8)	0.708
Fat intake (g/d)	112.6 (52.1)	107.9 (49.1)	117.4 (55.0)	0.399
Carbohydrate/sugar intake (g/d)	239.9 (79.9)	238.8 (72.9)	241.1 (87.0)	0.753
**Body composition**				
SMM (kg)	32.3 (7.4)	31.9 (7.7)	32.6 (7.3)	0.688
PBF (%)	37.5 (6.1)	37.6 (6.2)	37.5 (6.2)	0.888
BFM (kg)	34.1 (7.5)	33.8 (7.3)	34.4 (7.7)	0.748
VFA (cm^2^)	158.5 (36.4)	158.3 (36.4)	158.8 (36.8)	0.939
Waist circumference (cm)	102.6 (10.3)	102.7 (10.3)	102.4 (10.4)	0.941
Hip circumference (cm)	108.7 (6.6)	108.6 (7.5)	108.8 (5.7)	0.556
WHR	1.0 (0.1)	1.0 (0.1)	1.0 (0.0)	0.573
**Physical function and energy metabolism**				
HGS (kg)	37.1 (10.6)	37.3 (11.1)	36.8 (10.2)	0.859
FTSST (s)	8.6 (1.7)	8.6 (1.7)	8.7 (1.8)	0.870
Phase angle (°)	5.9 (0.6)	5.8 (0.6)	5.9 (0.6)	0.288
BMR (kcal/d)	1617.4 (258.5)	1616.9 (259.3)	1617.9 (260.4)	0.968
**Metabolic biomarkers**				
Fasting glucose (mmol/L)	5.1 (1.1)	5.3 (1.1)	5.0 (1.2)	0.167
Fasting insulin (μU/mL)	29.2 (18.9)	30.4 (19.5)	28.1 (18.3)	0.576
HOMA‐IR	0.8 (0.3)	0.8 (0.3)	0.7 (0.2)	0.430
Total cholesterol (mmol/L)	4.7 (0.8)	4.7 (0.9)	4.7 (0.7)	0.725
Triglycerides (mmol/L)	2.3 (1.6)	2.6 (2.1)	2.0 (0.9)	0.379
LDL‐C (mmol/L)	3.4 (0.9)	3.3 (0.9)	3.5 (0.8)	0.393
HDL‐C (mmol/L)	1.1 (0.2)	1.0 (0.2)	1.1 (0.2)	0.359
**Safety profile**				
SBP (mmHg)	135.5 (14.1)	136.0 (14.1)	135.1 (14.2)	0.345
DBP (mmHg)	88.0 (12.1)	88.5 (13.2)	87.6 (11.1)	0.578
ALT (U/L)	48.5 (33.7)	53.1 (40.4)	43.9 (24.9)	0.386
AST (U/L)	28.6 (15.2)	31.2 (19.4)	26.1 (8.9)	0.355
Albumin (g/L)	47.2 (2.0)	47.0 (2.0)	47.4 (2.1)	0.649
Creatinine (μmol/L)	73.0 (19.4)	72.3 (21.4)	73.6 (17.4)	0.673
Cystatin C (mg/L)	0.9 (0.2)	1.0 (0.2)	0.9 (0.1)	0.505
Uric acid (μmol/L)	421.2 (93.4)	426.5 (95.1)	415.8 (92.3)	0.730

Abbreviations: ALT, alanine aminotransferase; AST, aspartate aminotransferase; BFM, body fat mass; BMI, body mass index; BMR, basal metabolic rate; DBP, diastolic blood pressure; FTSST, five times sit‐to‐stand test; HDL‐C, high‐density lipoprotein cholesterol; HGS, handgrip strength; HOMA‐IR, homeostatic model assessment for insulin resistance; LDL‐C, low‐density lipoprotein cholesterol; PBF, body fat percentage; SBP, systolic blood pressure; SMM, Skeletal Muscle Mass; VFA, visceral fat area; WHR, waist‐hip ratio.

^a^ Data are presented as mean (SD) for continuous variables and *n* (%) for categorical variables.

^b^ Wilcoxon rank sum test; Pearson's chi‐squared test.

### 3.3 Primary Outcome: Skeletal Muscle Mass

**FIGURE 1:**
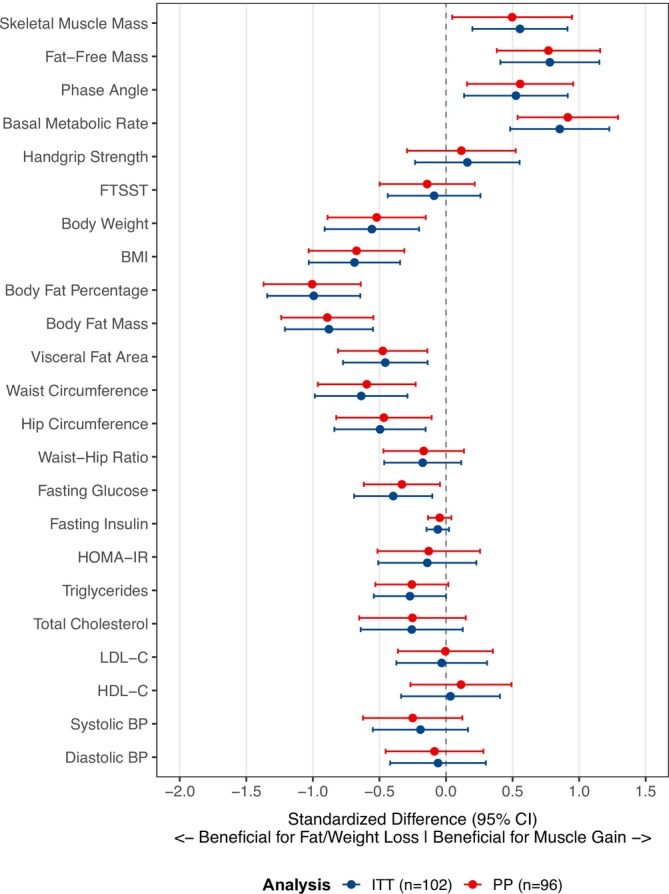
Effects of CaHMB‐enriched supplementation on skeletal muscle mass during weight loss. (A) Between‐group comparison of change in skeletal muscle mass (SMM) from baseline to week 12 in the ITT population (*n* = 102), shown as a raincloud plot. (B) Individual SMM trajectories from baseline to week 12 in the ITT population. (C) Time course of SMM at baseline, week 6 and week 12 in the ITT population (mean ± SE). (D–F) Corresponding between‐group comparison, individual trajectories and time course in the PP population (*n* = 96). In the figure, HMB denotes the CaHMB group. CaHMB, calcium beta‐hydroxy‐beta‐methylbutyrate; ITT, intention‐to‐treat; PP, per‐protocol; SE, standard error; SMM, skeletal muscle mass.

The primary analysis showed better preservation of muscle mass with the CaHMB‐enriched supplement during energy restriction. As shown in Figure 1A, the distribution of SMM changes differed significantly between groups, with the CaHMB group maintaining muscle mass (median change = +0.7 kg; IQR = −0.2 to 2.0) while the control group experienced significant muscle loss (median change = −0.6 kg; IQR = −1.9 to −0.1), resulting in a significant net benefit of 1.3 kg (95% CI = 0.5 to 1.9; *p* < 0.001, derived from ANCOVA Model 3 between‐group difference from baseline to week 12) with the CaHMB‐enriched supplement. Individual trajectory plots revealed a distinct pattern: most participants in the CaHMB group showed a stable or increasing SMM trend over 12 weeks, whereas a downward trend was prevalent in the control group (Figure 1B).

Time‐course analysis indicated that the divergence in SMM changes began as early as week 6 and widened by week 12 (Figure 1C). To assess clinical relevance, we performed a responder analysis defined by a gain of ≥ 0.5 kg in SMM. The proportion of responders was significantly higher in the CaHMB group compared with the control group (OR = 8.44; 95% CI = 3.23 to 25.22; *p* < 0.001) (Figure S2A). These findings were robust and consistent in the PP population (Figure 1D–F, Figure S2B and Table S1). Furthermore, the benefits of CaHMB‐enriched supplementation were sustained across unadjusted, minimally adjusted and fully adjusted statistical models (Table S2), and sensitivity analyses regarding missing data assumptions confirmed the robustness of the primary findings (Table S3).

### 3.4 Body Composition and Muscle‐Fat Trade‐Off

**FIGURE 2:**
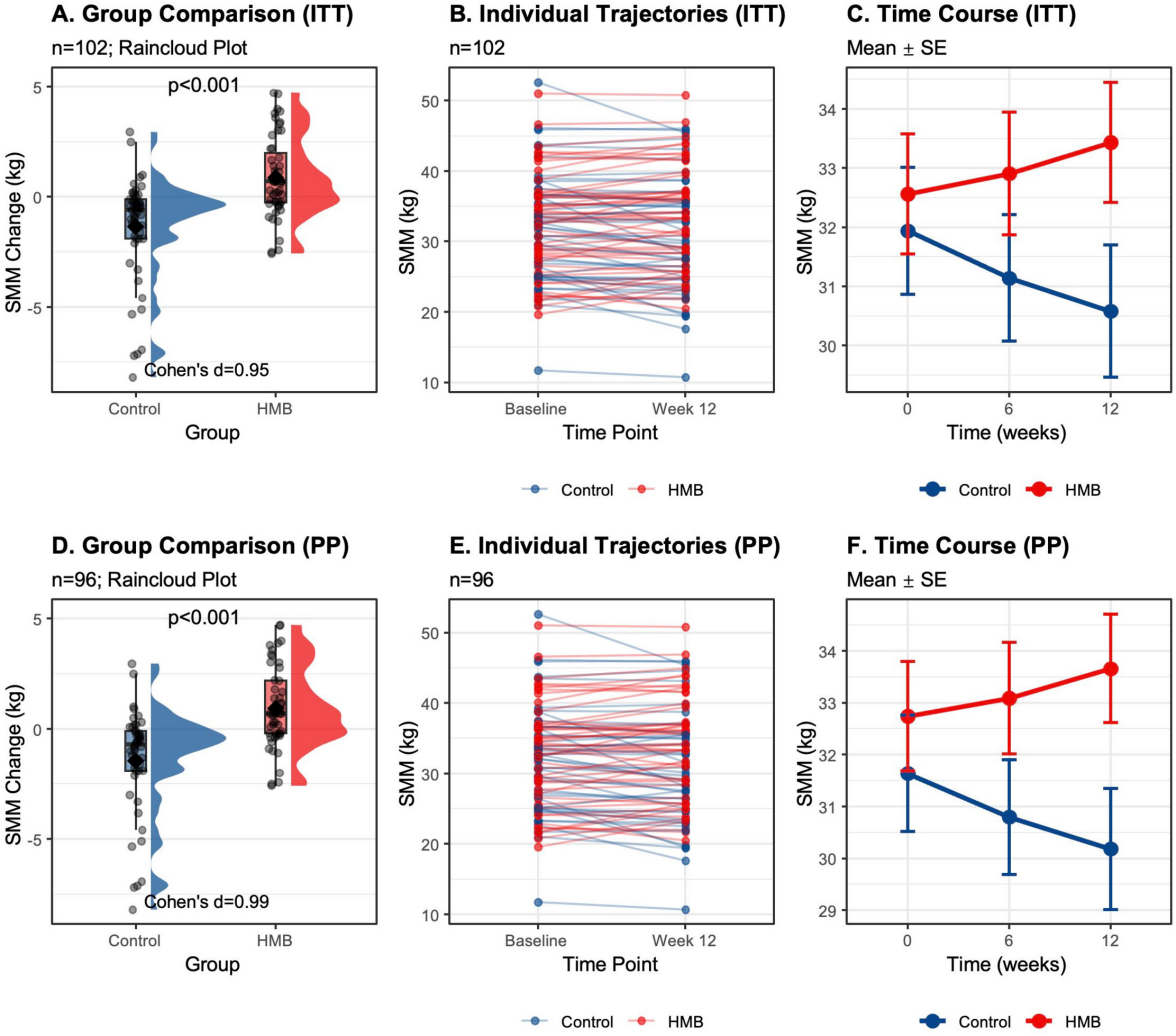
Comprehensive analysis of body‐composition changes and muscle‐fat trade‐off. (A) Muscle‐fat trade‐off plot in the ITT population, with change in SMM on the x‐axis and change in visceral fat area (VFA) on the y‐axis. Points represent individual participants, and diamonds represent group means. The lower‐right quadrant indicates the ideal zone of gaining or preserving muscle while losing visceral fat. (B) Heatmap of standardized effect sizes (Cohen's d) for key body‐composition indicators in the ITT and PP populations. Red indicates a beneficial effect for SMM gain, and blue indicates a beneficial effect for reduction in adiposity measures. (C) Raincloud plot of VFA change in the ITT population. (D) Individual VFA trajectories from baseline to week 12. (E) Time course of VFA at baseline, week 6 and week 12 (mean ± SE). (F) Exploratory responder analysis for VFA reduction, defined as a decrease of at least 20 cm^2^. In the figure, HMB denotes the CaHMB group. CaHMB, calcium beta‐hydroxy‐beta‐methylbutyrate; ITT, intention‐to‐treat; PBF, percent body fat; PP, per‐protocol; SE, standard error; SMM, skeletal muscle mass; VFA, visceral fat area.

We further explored the comprehensive changes in body composition. Figure 2A illustrates the muscle‐fat trade‐off, where individual changes in SMM are plotted against changes in VFA. A substantial proportion of CaHMB participants clustered in the lower‐right quadrant, representing the favourable outcome of simultaneous muscle gain and visceral fat loss. In contrast, control participants were largely distributed in the lower‐left quadrant, indicating concurrent loss of both muscle and visceral fat.

The heatmap of standardized effect sizes (Cohen's *d*) provided a holistic view of the intervention's impact (Figure 2B). The CaHMB‐enriched supplement showed medium‐to‐large beneficial effects on SMM retention and VFA reduction, with consistent directions in both ITT and PP analyses. Specifically for VFA, the CaHMB group achieved a significantly greater reduction compared with the control group (Figure 2C), with individual slopes showing a steeper decline (Figure 2D). This pattern was evident throughout the intervention period (Figure 2E). The exploratory responder analysis for VFA reduction ≥ 20 cm^2^ was directionally consistent with greater visceral fat reduction in the CaHMB group but did not meet conventional statistical significance (Figure 2F).

### 3.5 Summary of Treatment Effects and Ancillary Analyses

**FIGURE 3:**
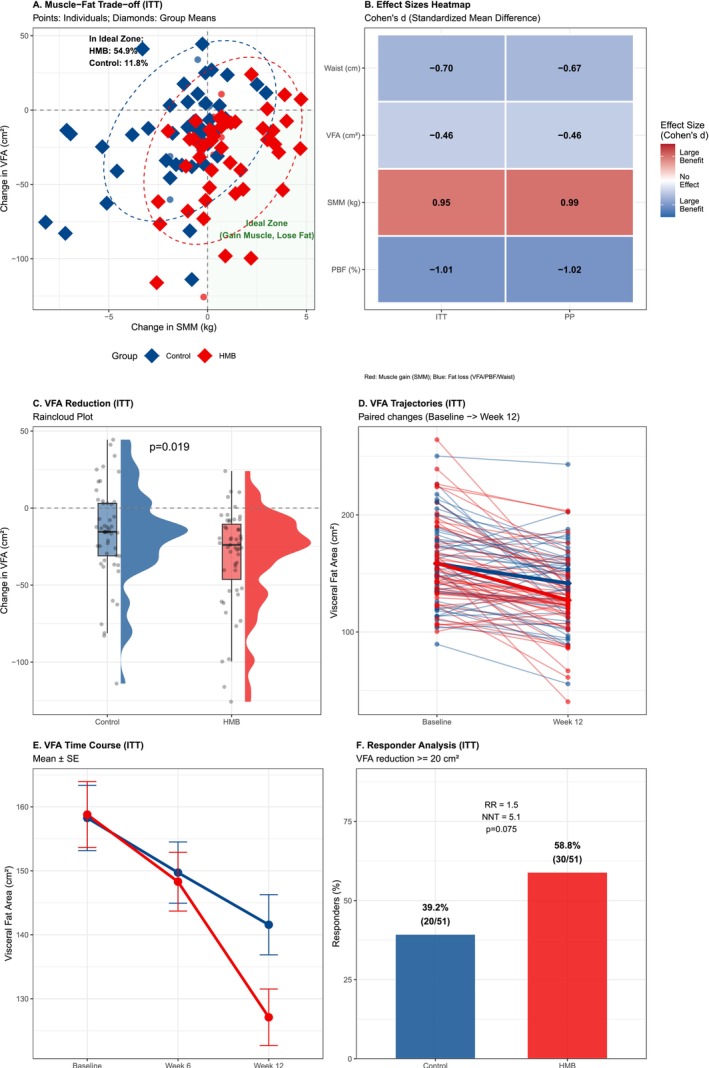
Forest plot of standardized treatment effects across body‐composition, functional, metabolic and safety‐related outcomes. Standardized differences with 95% CIs are shown for the ITT population (blue; *n* = 102) and PP population (red; *n* = 96). Positive values indicate greater increases and negative values indicate greater reductions in the CaHMB group relative to the control group on the original outcome scale; therefore, the clinically beneficial direction differs by outcome (e.g., positive for SMM, FFM and BMR and negative for adiposity measures, FTSST and most cardiometabolic markers). In the figure, HMB denotes the CaHMB group. BFM, body fat mass; BMI, body mass index; BMR, basal metabolic rate; CaHMB, calcium beta‐hydroxy‐beta‐methylbutyrate; CI, confidence interval; DBP, diastolic blood pressure; FFM, fat‐free mass; FTSST, five times sit‐to‐stand test; HDL‐C, high‐density lipoprotein cholesterol; HGS, handgrip strength; HOMA‐IR, homeostatic model assessment for insulin resistance; ITT, intention‐to‐treat; LDL‐C, low‐density lipoprotein cholesterol; PBF, percent body fat; PP, per‐protocol; SBP, systolic blood pressure; SE, standard error; SMM, skeletal muscle mass; TC, total cholesterol; TG, triglycerides; VFA, visceral fat area; WHR, waist‐to‐hip ratio.

Detailed treatment effects on the primary and selected secondary outcomes are presented in Table 2. Figure 3 summarizes standardized treatment effects across body‐composition, functional, metabolic and safety‐related outcomes. After full adjustment for baseline covariates and changes in diet and physical activity (Model 3), the primary treatment effect favoured the CaHMB group for SMM (adjusted median difference 1.3 kg; 95% CI = 0.5 to 1.9). Exploratory secondary analyses showed greater reductions in BFM (AMD −4.9 kg; 95% CI = −6.8 to −3.1) and VFA (AMD −14.9 cm^2^; 95% CI = −25.1 to −4.5; *p* = 0.004). Detailed distributional changes for secondary body composition and physical function outcomes are shown in Figure S3, while metabolic biomarker changes are detailed in Figure S4. No significant between‐group differences were observed in insulin resistance (HOMA‐IR) or lipid profiles compared with controls.

**TABLE 2:** Summary of primary and secondary body composition and metabolic outcomes (ITT population, *n* = 102).

	Outcome	CaHMB group Change (mean ± SD or median [IQR])	Control group Change (mean ± SD or median [IQR])	Difference (mean or median)	95% CI	*p*
Primary outcome	Skeletal muscle mass (kg)	0.7 (−0.2 to 2.0)	−0.6 (−1.9 to −0.1)	1.3	(0.5, 1.9)	< 0.001
Body composition	VFA (cm^2^)	−31.7 ± 31.9	−16.7 ± 31.6	−14.9	(−25.1, −4.5)	0.004
	Weight (kg)	−5.9 ± 5.9	−2.4 ± 4.9	−3.1	(−5.1, −1.1)	0.002
	BMI (kg/m^2^)	−2.3 ± 2.5	−0.7 ± 1.6	−1.6	(−2.3, −0.8)	< 0.001
	Body fat mass (kg)	−6.6 ± 5.8	−1.5 ± 4.0	−4.9	(−6.8, −3.1)	< 0.001
	Body fat percentage (%)	−5.3 ± 4.7	−0.5 ± 3.4	−4.7	(−6.4, −3.0)	< 0.001
Muscle quality and function	Basal metabolic rate (kcal/d)	42.0 ± 73.3	−17.0 ± 59.0	62.0	(35.0, 89.0)	< 0.001
	Phase angle (°)	0.1 ± 0.4	−0.1 ± 0.4	0.2	(0.1, 0.4)	0.009
	Handgrip strength (kg)	1.6 ± 4.5	0.9 ± 4.2	0.7	(−1.0, 2.4)	0.420
Metabolic biomarkers	Fasting glucose (mmol/L)	0.2 ± 0.9	0.6 ± 1.3	−0.4	(−0.8, −0.1)	0.009
	HOMA‐IR	−0.3 (−0.4 to −0.1)	−0.2 (−0.4 to −0.0)	−0.1	(−0.2, 0.0)	0.228
	Total cholesterol (mmol/L)	−0.3 ± 0.7	−0.2 ± 0.8	−0.2	(−0.5, 0.1)	0.186
	Triglycerides (mmol/L)	0.1 (−0.4 to 0.6)	0.3 (−0.3 to 1.0)	−0.3	(−0.7, 0.2)	0.322
	LDL‐C (mmol/L)	−1.4 ± 0.8	−1.3 ± 0.9	−0.0	(−0.3, 0.3)	0.851
	HDL‐C (mmol/L)	0.1 ± 0.1	0.1 ± 0.2	0.0	(−0.1, 0.1)	0.856
	Systolic BP (mmHg)	−3.8 ± 15.8	−1.6 ± 14.1	−2.9	(−8.2, 2.5)	0.290
	Diastolic BP (mmHg)	2.0 ± 12.7	2.4 ± 13.1	−0.8	(−5.4, 3.8)	0.742

*Note:* Data are presented as mean ± SD or median (IQR) for nonnormally distributed variables (SMM, triglycerides, insulin and HOMA‐IR). Differences, 95% CIs and *p* values are derived from ANCOVA (Model 3) for normal variables. For nonnormal variables, differences are simple median differences with bootstrap 95% CIs, and *p* values are from quantile regression or Wilcoxon rank‐sum tests, as prespecified for distributional assumptions.

Abbreviation: ITT, intention‐to‐treat population.

**FIGURE 4:**
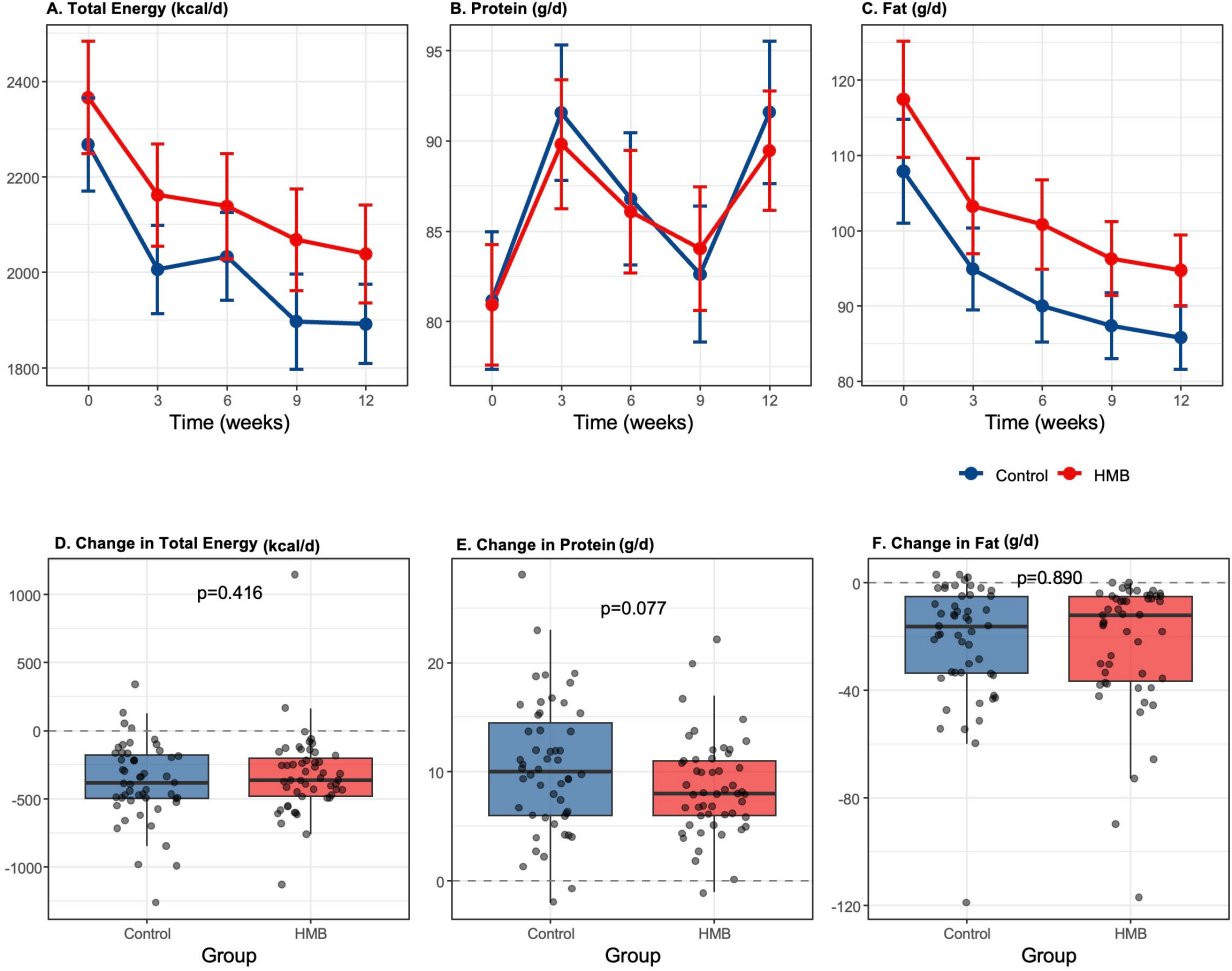
Dietary intake trajectories during the 12‐week intervention. (A–C) Longitudinal trajectories of total energy intake, dietary protein intake and dietary fat intake at baseline and weeks 3, 6, 9 and 12 (mean ± SE). (D‐F) Boxplots showing changes from baseline to week 12 in total energy intake, dietary protein intake and dietary fat intake. No significant between‐group differences were observed in these background dietary parameters. In the figure, HMB denotes the CaHMB group. CaHMB, calcium beta‐hydroxy‐beta‐methylbutyrate; SE, standard error.

Longitudinal monitoring of dietary intake and physical activity showed no significant between‐group differences in total dietary energy, protein (excluding supplements) or fat (Figure 4A–F), nor in physical activity levels (IPAQ scores) at any time point during the 12 weeks (Figure S5).

Exploratory subgroup analyses showed that the primary SMM treatment effect was directionally consistent across strata of age (< 40 vs. ≥ 40 years), gender (male vs. female) and baseline BMI (< 32 vs. ≥ 32 kg/m^2^), with no significant interactions detected (Figure S6). Exploratory mediation analyses generated hypotheses that maintenance of BMR and improvements in phase angle may partly explain the observed SMM preservation (Figure S7).

### 3.6 Safety

The intervention was safe and well tolerated. No adverse events were reported in either group throughout the study. Furthermore, no significant differences were observed between the CaHMB and control groups in changes in vital signs (systolic/diastolic blood pressure) (Table 2) or laboratory safety parameters (liver enzymes, renal function and blood counts) from baseline to week 12 (Figure S8).

## 4 Discussion

In this randomized, double‐blind, controlled trial, CaHMB‐enriched nutritional supplementation preserved SMM and enhanced visceral fat loss in adults with obesity undergoing dietary energy restriction. The primary ITT analysis showed a between‐group difference of 1.3 kg in SMM (95% CI = 0.5 to 1.9; *p* < 0.001), with a large effect size (Cohen's *d* = 0.84). The CaHMB group also achieved greater VFA reduction (AMD −14.9 cm^2^; *p* = 0.004), indicating a favourable muscle‐fat profile rather than weight loss driven by parallel losses of fat and skeletal muscle.

Mechanistically, CaHMB may support muscle protein metabolism by increasing muscle protein synthesis through mTORC1/p70S6K signalling and by reducing protein degradation through inhibition of ubiquitin‐proteasome pathways [11, 23]. CaHMB may also contribute to sarcolemmal membrane integrity through the mevalonate pathway, which is consistent with the observed improvement in phase angle. The 1.3 kg between‐group difference in SMM is clinically relevant because it is comparable to the annual magnitude of muscle loss reported in sarcopenia‐related contexts, although direct functional benefit should be tested in longer trials.

Our findings add to the current literature on CaHMB by extending prior evidence from older adults with sarcopenia [20], exercise‐induced muscle damage [24] and nutritional strategies during weight loss [25] to a sedentary adult population with obesity undergoing dietary energy restriction. In contrast to conventional weight‐loss interventions, where lean mass commonly accounts for a clinically meaningful proportion of total weight loss [25], the control group in this trial lost SMM whereas the CaHMB group maintained or gained SMM. The magnitude of the muscle‐sparing effect was similar to the range reported for resistance training interventions in adults with overweight or obesity [26].

The interpretation of this effect must consider the composition of the intervention supplement. The CaHMB supplement provided 24 g/day of protein plus other nutritional components, whereas the control supplement contained maltodextrin without protein or CaHMB. Although background dietary protein intake excluding the intervention supplement was comparable between groups throughout the study, the current design cannot isolate CaHMB‐specific effects from the protein‐containing supplement matrix. The findings therefore support the clinical efficacy of the complete CaHMB‐enriched nutritional package, while factorial trials are needed to separate independent CaHMB effects from protein‐mediated or synergistic effects.

These results are relevant in the era of potent antiobesity pharmacotherapy. Recent obesity pharmacotherapy studies have raised concern that substantial weight loss may be accompanied by loss of lean mass [3, 6]. Our data suggest that CaHMB‐enriched nutritional supplementation may help preserve skeletal muscle during dietary energy restriction, but this trial did not test combination therapy with GLP‐1 receptor agonists. Combination strategies pairing pharmacological weight loss with nutritional muscle protection should therefore be considered hypothesis‐generating and require direct evaluation in future randomized trials.

The exploratory mediation analyses generated hypotheses for two plausible pathways linking CaHMB‐enriched supplementation with SMM preservation: maintenance of BMR and improvement in phase angle. Because skeletal muscle is a major determinant of resting energy expenditure, preserving muscle mass may reduce adaptive thermogenesis during weight loss [27]. Phase angle reflects cellular membrane integrity and tissue quality, and its improvement is consistent with the proposed membrane‐stabilizing actions of CaHMB. The greater VFA reduction observed in the CaHMB group may also contribute to metabolic risk reduction, although insulin resistance and lipid outcomes did not differ significantly between groups in this 12‐week trial. In addition, no significant between‐group differences were observed for handgrip strength or the five times sit‐to‐stand test, so short‐term functional benefit cannot be inferred from these body‐composition changes.

The concept of a muscle‐fat trade‐off is clinically important because current obesity frameworks emphasize adiposity distribution, function and health consequences rather than body weight alone [7, 13]. The responder analysis showed that the CaHMB‐enriched supplement increased the likelihood of gaining at least 0.5 kg of SMM during weight loss. Participants were informed only that they would receive a nutritional supplement for weight management, and instructions were delivered identically to both groups. Comparable physical activity and background dietary intake across follow‐up suggest that supplement‐related expectations did not differentially influence behaviour, although formal assessment of participant beliefs was not performed.

## 5 Limitations

Several limitations must be acknowledged. First, deviations from the original protocol occurred. The recruitment target was expanded from 72 to 102 participants to enhance statistical power for subgroup analyses. Additionally, due to COVID‐19–related restrictions during the study period, some follow‐up visits had a widened time window (±5 days instead of ±3 days), and dietary counselling was partially shifted to online video consultations. While these deviations were necessary adaptations, they were applied equally to both groups and are unlikely to have biased the treatment effect. Second, body composition was assessed using multifrequency BIA rather than dual‐energy x‐ray absorptiometry (DXA) or MRI. Although the InBody 770 shows high correlation with DXA [16], BIA can be influenced by hydration status. We minimized this error by strictly standardizing measurement conditions (fasting and specific time of day). Third, the 12‐week duration, while sufficient to detect physiological changes, does not allow us to assess long‐term weight maintenance, durability of the muscle‐protective effect or delayed functional benefits. Fourth, the choice of a protein‐free maltodextrin control supplement limits our ability to definitively disentangle CaHMB‐specific effects from the protein and micronutrients provided in the intervention package. Although background dietary energy and macronutrient intakes were monitored and appeared broadly comparable between groups, residual dietary confounding cannot be excluded. Finally, a formal blinding assessment was not conducted, molecular biomarkers of muscle metabolism (e.g., IGF‐1) were not measured, and secondary, responder, subgroup and mediation analyses were not adjusted for multiplicity and should be interpreted as exploratory.

## 6 Future Directions

Future research should prioritize multiarm factorial trials that isolate CaHMB, protein and their combined effects; longer interventions of at least 6 months to assess durability; studies using DXA or MRI with molecular biomarkers such as IGF‐1, myostatin and inflammatory markers; and trials testing CaHMB‐enriched supplementation alongside contemporary antiobesity pharmacotherapy. These designs would clarify whether the body‐composition benefits observed here translate into better physical function, metabolic outcomes and long‐term weight maintenance.

## 7 Conclusions

In this randomized trial of Chinese adults with obesity undergoing dietary energy restriction, CaHMB‐enriched nutritional supplementation preserved skeletal muscle mass and produced greater visceral fat and body fat reductions than an energy‐matched maltodextrin control. The findings support CaHMB‐enriched supplementation as a potential nutritional adjunct for improving the quality of weight loss, while longer‐term and factorial trials are needed before making CaHMB‐specific clinical recommendations.

## Funding

This study was funded by the K&D Program of the Sichuan Science and Technology Department (Grant No. 2020YFS0573). The funder of the study had no role in study design, data collection, data analysis, data interpretation or writing of the report. The corresponding authors had full access to all the data in the study and had final responsibility for the decision to submit for publication.

## Conflicts of Interest

The authors declare no conflicts of interest.

## Supporting information




**Table S1:** Summary of primary and secondary outcomes in the per‐protocol population (*n* = 96).
**Table S2:** Comparison of treatment effects across statistical models in ITT and PP populations.
**Table S3:** Sensitivity analysis for missing data.
**Figure S1:** Trial profile (CONSORT flowchart).
**Figure S2:** Effects of CaHMB supplementation on skeletal muscle mass during weight loss.
**Figure S3:** Detailed changes in body composition and physical function.
**Figure S4:** Detailed changes in metabolic biomarkers.
**Figure S5:** Physical activity trajectories.
**Figure S6:** Subgroup analyses of the primary outcome.
**Figure S7:** Mediation analysis.
**Figure S8:** Safety evaluation.

## Data Availability

The analysis code is provided as Supporting Information. Deidentified individual participant data are not publicly deposited because the ethics approval and informed consent for this trial did not include unrestricted public sharing of participant‐level data, and the dataset will also be used for planned secondary analyses. Deidentified data supporting the findings may be made available from the corresponding author upon reasonable request by qualified researchers, subject to institutional approval and, where applicable, a data use agreement. No individual‐level participant data are included in the supplementary analysis code package.
