# Supplementary material for: Calcium Beta‐Hydroxy‐Beta‐Methylbutyrate‐Enriched Nutritional Supplementation During Dietary Weight Loss in Adults With Obesity: A Randomized Controlled Trial
Source: J Cachexia Sarcopenia Muscle. 2026 Jul 13;17(4):e70343. doi: 10.1002/jcsm.70343 (PMC13365355; doi:10.1002/jcsm.70343)
Supplement: Supplementary file 1 — Table S1: Summary of primary and secondary outcomes in the per‐protocol population (n = 96). Table S2: Comparison of treatment effects across statistical models in ITT and PP populations. Table S3: Sensitivity analysis for missing data. Figure S1: Trial profile (CONSORT flowchart). Figure S2: Effects of CaHMB supplementation on skeletal muscle mass during weight loss. Figure S3: Detailed changes in body composition and physical function. Figure S4: Detailed changes in metabolic biomarkers. Figure S5: Physical activity trajectories. Figure S6: Subgroup analyses of the primary outcome. Figure S7: Mediation analysis. Figure S8: Safety evaluation. [file JCSM-17-e70343-s001.docx]

# Supplementary Appendix

**Manuscript Title: Calcium beta-hydroxy-beta-methylbutyrate-enriched nutritional supplementation during dietary weight loss in adults with obesity: a randomized controlled trial**

**Authors:** Jiaojiao Jiang, Yuxiang Liang, Renjie Wang, Wenhua Jiang, Xiaofan Jing, Ming Yang

## Table of Contents

| Item | Title | Page |
| --- | --- | --- |
| **Supplementary Tables** | |  |
| Table S1 | Summary of Primary and Secondary Outcomes in the Per-Protocol Population (n=96) | 3 |
| Table S2 | Comparison of Treatment Effects Across Statistical Models in ITT and PP Populations | 5 |
| Table S3 | Sensitivity Analysis for missing data | 7 |
| **Supplementary Figures** | |  |
| Figure S1 | Trial Profile (CONSORT Flowchart) | 8 |
| Figure S2 | Effects of CaHMB supplementation on skeletal muscle mass during weight loss | 9 |
| Figure S3 | Detailed changes in body composition and physical function | 10 |
| Figure S4 | Detailed changes in metabolic biomarkers | 12 |
| Figure S5 | Physical activity trajectories | 13 |
| Figure S6 | Subgroup analyses of the primary outcome | 14 |
| Figure S7 | Mediation analysis | 15 |
| Figure S8 | Safety evaluation | 16 |

## Supplementary Tables

| **Table S1. Summary of Primary and Secondary Outcomes in the Per-Protocol Population (n=96)** | | | | | | |
| --- | --- | --- | --- | --- | --- | --- |
|  | **Outcome** | CaHMB Group (n=49) Mean Change (Mean ± SD or Median [IQR]) | **Control Group (n=47) Mean Change (Mean ± SD or Median [IQR])** | **Difference (Mean or Median)** | **95% CI** | **P value** |
| Primary Outcome | Skeletal Muscle Mass (kg) | 0.7 (-0.2 to 2.2) | -0.7 (-1.9 to -0.1) | 1.4 | (0.7, 2.1) | <0.001 |
| Body Composition | Visceral Fat Area (cm²) | -32.3 ± 32.4 | -18.3 ± 31.4 | -15.4 | (-26.4, -4.5) | 0.006 |
|  | Body Weight (kg) | -6.0 ± 5.9 | -2.6 ± 4.9 | -3.3 | (-5.5, -1.1) | 0.004 |
|  | BMI (kg/m²) | -2.4 ± 2.5 | -0.7 ± 1.6 | -1.7 | (-2.5, -0.8) | <0.001 |
|  | Body Fat Mass (kg) | -6.7 ± 5.8 | -1.5 ± 4.1 | -5.2 | (-7.3, -3.2) | <0.001 |
|  | Body Fat Percentage (%) | -5.3 ± 4.7 | -0.5 ± 3.5 | -4.9 | (-6.6, -3.2) | <0.001 |
| Muscle Quality & Function | Basal Metabolic Rate (kcal/d) | 43.5 ± 74.4 | -17.9 ± 56.9 | 61.4 | (34.4, 88.3) | <0.001 |
|  | Phase Angle (°) | 0.1 ± 0.4 | -0.1 ± 0.4 | 0.2 | (0.1, 0.4) | 0.006 |
|  | Handgrip Strength (kg) | 1.6 ± 4.6 | 1.1 ± 4.2 | 0.5 | (-1.3, 2.3) | 0.566 |
| Metabolic Biomarkers | Fasting Glucose (mmol/L) | 0.2 ± 0.9 | 0.5 ± 1.2 | -0.2 | (-0.7, 0.2) | 0.265 |
|  | HOMA-IR | -0.3 (-0.4 to -0.1) | -0.2 (-0.4 to -0.1) | -0.1 | (-0.2, 0.0) | 0.435 |
|  | Total Cholesterol (mmol/L) | -0.3 ± 0.7 | -0.2 ± 0.8 | -0.1 | (-0.4, 0.2) | 0.406 |
|  | Triglycerides (mmol/L) | 0.0 (-0.4 to 0.5) | 0.3 (-0.3 to 1.0) | -0.3 | (-0.7, 0.2) | 0.358 |
|  | LDL-C (mmol/L) | -1.3 ± 0.8 | -1.3 ± 0.9 | -0.1 | (-0.4, 0.3) | 0.686 |
|  | HDL-C (mmol/L) | 0.1 ± 0.1 | 0.1 ± 0.2 | 0.0 | (-0.1, 0.1) | 0.920 |
|  | Systolic BP (mmHg) | -4.0 ± 15.8 | -1.0 ± 14.0 | -3.0 | (-9.0, 3.1) | 0.332 |
|  | Diastolic BP (mmHg) | 1.9 ± 11.7 | 2.7 ± 13.4 | -0.8 | (-5.9, 4.3) | 0.752 |
| Data are presented as Mean ± SD or Median (IQR) for non-normally distributed variables (SMM, VFA, Triglycerides, Insulin, HOMA-IR). Differences, 95% CIs, and P-values are derived from ANCOVA (Model 3) for normal variables. For non-normal variables, differences are simple median differences with Bootstrap 95% CIs, and P-values are from Wilcoxon rank sum tests. PP: Per-Protocol population. | | | | | | |

| **Table S2: Comparison of Treatment Effects Across Statistical Models in ITT and PP Populations** | | | | | | |
| --- | --- | --- | --- | --- | --- | --- |
| Outcome | Analysis Set | Model 1 (Unadjusted) β (95% CI) | Model 2 (Minimally Adjusted) β (95% CI) | Model 3 (Fully Adjusted) β (95% CI) | P-value* |  |
| Skeletal Muscle Mass (kg) | ITT Population (n=102) | 1.30 (0.61, 1.99) | 1.20 (0.50, 1.90) | 1.33 (0.45, 2.20) | <0.001 |  |
|  | PP Population (n=96) | 1.40 (0.67, 2.13) | 1.31 (0.51, 2.11) | 1.22 (0.20, 2.24) | 0.019 |  |
| Visceral Fat Area (cm²) | ITT Population (n=102) | -14.99 (-27.45, -2.53) | -14.74 (-25.18, -4.30) | -14.79 (-25.10, -4.49) | 0.005 |  |
|  | PP Population (n=96) | -13.97 (-26.91, -1.04) | -14.89 (-25.82, -3.97) | -15.45 (-26.36, -4.53) | 0.006 |  |
| Body Fat Percentage (%) | ITT Population (n=102) | -4.76 (-6.38, -3.15) | -4.76 (-6.38, -3.14) | -4.70 (-6.36, -3.05) | <0.001 |  |
|  | PP Population (n=96) | -4.86 (-6.55, -3.17) | -4.85 (-6.55, -3.14) | -4.84 (-6.60, -3.08) | <0.001 |  |
| Body Weight (kg) | ITT Population (n=102) | -3.49 (-5.61, -1.37) | -3.26 (-5.19, -1.33) | -3.14 (-5.14, -1.14) | 0.002 |  |
|  | PP Population (n=96) | -3.32 (-5.53, -1.11) | -3.03 (-5.05, -1.02) | -2.96 (-5.05, -0.86) | 0.006 |  |
| BMI (kg/m²) | ITT Population (n=102) | -1.69 (-2.52, -0.87) | -1.62 (-2.38, -0.85) | -1.55 (-2.33, -0.78) | <0.001 |  |
|  | PP Population (n=96) | -1.65 (-2.52, -0.79) | -1.60 (-2.40, -0.80) | -1.54 (-2.36, -0.71) | <0.001 |  |
| Body Fat Mass (kg) | ITT Population (n=102) | -5.16 (-7.12, -3.20) | -5.01 (-6.85, -3.17) | -4.92 (-6.77, -3.07) | <0.001 |  |
|  | PP Population (n=96) | -5.22 (-7.27, -3.17) | -5.15 (-7.08, -3.22) | -5.06 (-7.02, -3.10) | <0.001 |  |
| Basal Metabolic Rate (kcal) | ITT Population (n=102) | 58.93 (32.79, 85.06) | 58.95 (32.83, 85.08) | 61.99 (35.00, 88.98) | <0.001 |  |
|  | PP Population (n=96) | 61.37 (34.45, 88.28) | 61.94 (35.13, 88.76) | 66.71 (39.22, 94.21) | <0.001 |  |
| Phase Angle (°) | ITT Population (n=102) | 0.24 (0.07, 0.41) | 0.22 (0.05, 0.40) | 0.23 (0.06, 0.41) | 0.009 |  |
|  | PP Population (n=96) | 0.25 (0.07, 0.42) | 0.23 (0.06, 0.41) | 0.25 (0.07, 0.43) | 0.007 |  |
| Handgrip Strength (kg) | ITT Population (n=102) | 0.67 (-1.04, 2.38) | 0.67 (-1.05, 2.39) | 0.70 (-1.01, 2.40) | 0.420 |  |
|  | PP Population (n=96) | 0.51 (-1.26, 2.29) | 0.52 (-1.26, 2.30) | 0.50 (-1.28, 2.29) | 0.575 |  |
| Five Times Sit-to-Stand (s) | ITT Population (n=102) | -0.19 (-1.06, 0.67) | -0.14 (-0.87, 0.60) | -0.19 (-0.96, 0.57) | 0.612 |  |
|  | PP Population (n=96) | -0.30 (-1.20, 0.61) | -0.19 (-0.97, 0.58) | -0.32 (-1.11, 0.48) | 0.434 |  |
| Fasting Glucose (mmol/L) | ITT Population (n=102) | -0.35 (-0.79, 0.09) | -0.49 (-0.83, -0.14) | -0.45 (-0.78, -0.12) | 0.009 |  |
|  | PP Population (n=96) | -0.24 (-0.67, 0.19) | -0.38 (-0.69, -0.07) | -0.35 (-0.65, -0.05) | 0.024 |  |
| Fasting Insulin (μU/mL) | ITT Population (n=102) | -3.40 (-21.52, 14.73) | -4.93 (-22.47, 12.61) | -4.38 (-22.39, 13.63) | 0.631 |  |
|  | PP Population (n=96) | 0.16 (-17.92, 18.25) | -1.93 (-19.39, 15.53) | -0.68 (-18.79, 17.43) | 0.941 |  |
| HOMA-IR | ITT Population (n=102) | -0.08 (-0.24, 0.08) | -0.11 (-0.25, 0.04) | -0.11 (-0.26, 0.04) | 0.166 |  |
|  | PP Population (n=96) | -0.04 (-0.20, 0.12) | -0.08 (-0.23, 0.07) | -0.08 (-0.23, 0.08) | 0.326 |  |
| Total Cholesterol (mmol/L) | ITT Population (n=102) | -0.12 (-0.41, 0.17) | -0.14 (-0.41, 0.14) | -0.19 (-0.47, 0.09) | 0.186 |  |
|  | PP Population (n=96) | -0.12 (-0.42, 0.17) | -0.13 (-0.41, 0.16) | -0.18 (-0.48, 0.11) | 0.215 |  |
| Triglycerides (mmol/L) | ITT Population (n=102) | -0.47 (-1.30, 0.36) | -0.54 (-1.39, 0.30) | -0.58 (-1.45, 0.29) | 0.189 |  |
|  | PP Population (n=96) | -0.48 (-1.36, 0.40) | -0.56 (-1.45, 0.32) | -0.66 (-1.58, 0.26) | 0.156 |  |
| LDL-C (mmol/L) | ITT Population (n=102) | -0.09 (-0.42, 0.23) | 0.01 (-0.27, 0.28) | -0.03 (-0.31, 0.26) | 0.851 |  |
|  | PP Population (n=96) | -0.07 (-0.41, 0.27) | 0.03 (-0.27, 0.32) | -0.00 (-0.30, 0.30) | 0.980 |  |
| HDL-C (mmol/L) | ITT Population (n=102) | -0.01 (-0.07, 0.05) | 0.01 (-0.05, 0.06) | 0.01 (-0.05, 0.06) | 0.856 |  |
|  | PP Population (n=96) | 0.00 (-0.06, 0.06) | 0.01 (-0.04, 0.07) | 0.02 (-0.04, 0.07) | 0.556 |  |
| Systolic BP (mmHg) | ITT Population (n=102) | -2.20 (-8.08, 3.68) | -2.59 (-7.92, 2.74) | -2.86 (-8.21, 2.48) | 0.290 |  |
|  | PP Population (n=96) | -2.98 (-9.04, 3.09) | -3.38 (-8.88, 2.11) | -3.73 (-9.31, 1.84) | 0.187 |  |
| Diastolic BP (mmHg) | ITT Population (n=102) | -0.39 (-5.45, 4.68) | -0.81 (-5.25, 3.63) | -0.77 (-5.38, 3.85) | 0.742 |  |
|  | PP Population (n=96) | -0.81 (-5.89, 4.27) | -1.23 (-5.61, 3.15) | -1.07 (-5.66, 3.51) | 0.644 |  |
| * P-value for Model 3. | | | | | | |
| Model 1: Unadjusted. | | | | | | |
| Model 2: Adjusted for baseline value of the outcome. | | | | | | |
| Model 3: Adjusted for baseline value, Age, Sex, Change in Dietary Energy, Change in Dietary Protein, and Change in Physical Activity (IPAQ). | | | | | | |
| Abbreviations: ITT, Intent-to-Treat; PP, Per-Protocol; CI, Confidence Interval. | | | | | | |

| **Table S3. Sensitivity Analysis for Missing Data (SMM Change)** | | | |
| --- | --- | --- | --- |
| Analysis Method | Effect Estimate (β) | 95% CI | P-value |
| Multiple Imputation (ITT, n=102) | 1.28 | (0.52, 2.05) | 0.001 |
| Complete Case Analysis (n=96) | 1.37 | (0.48, 2.26) | 0.003 |
| Median regression model adjusted for baseline SMM, age, and sex. | | | |
| Abbreviations: ITT, Intent-to-Treat; CI, Confidence Interval; SMM, Skeletal Muscle Mass. | | | |

## Supplementary Figures

### Figure S1


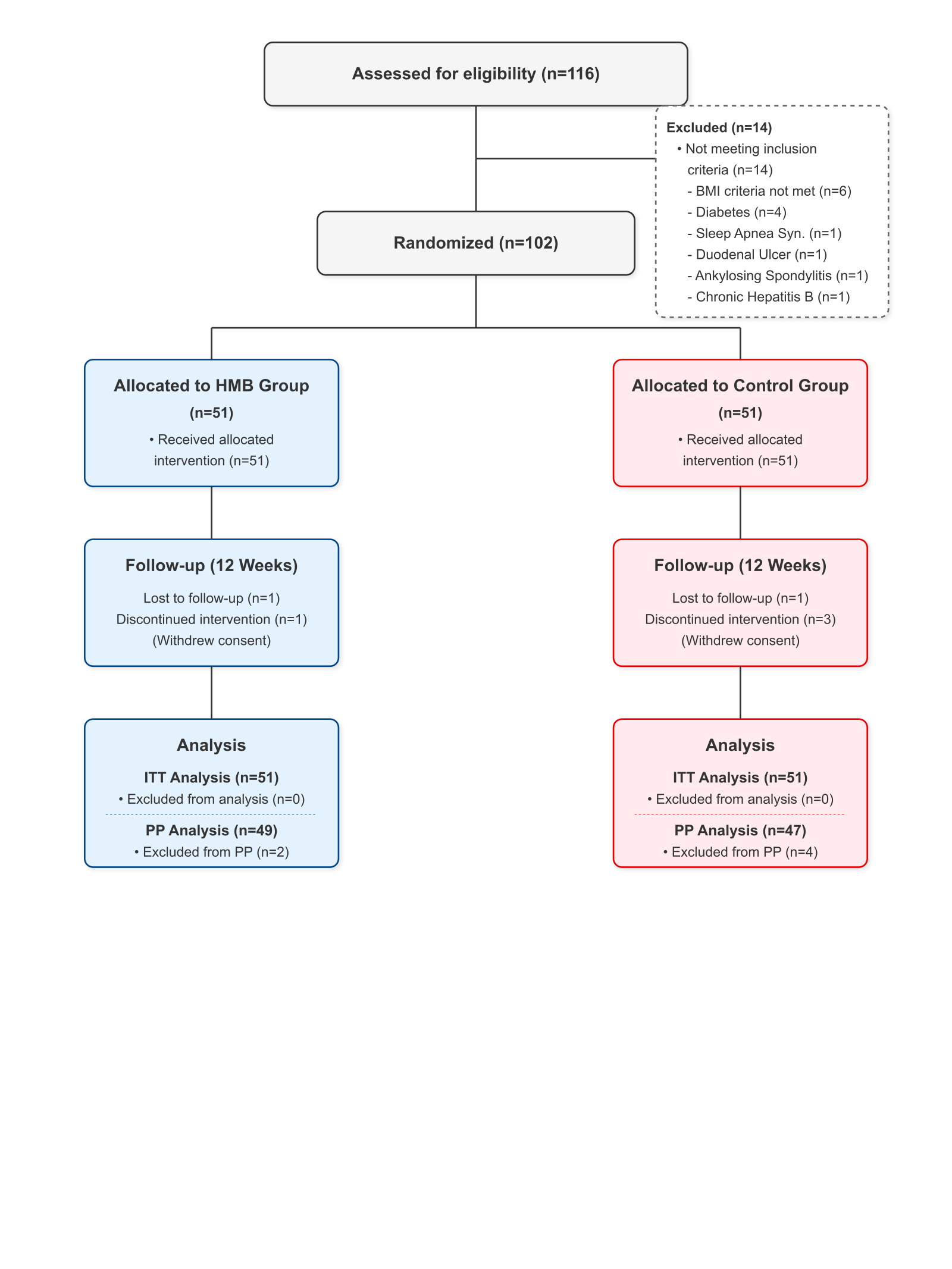


**Figure S1: Trial profile.**
Flowchart describing the screening, randomisation, allocation, follow-up, and analysis populations of the study, strictly adhering to CONSORT 2025 guidelines. ITT, Intention-to-Treat; PP, Per-Protocol.

###
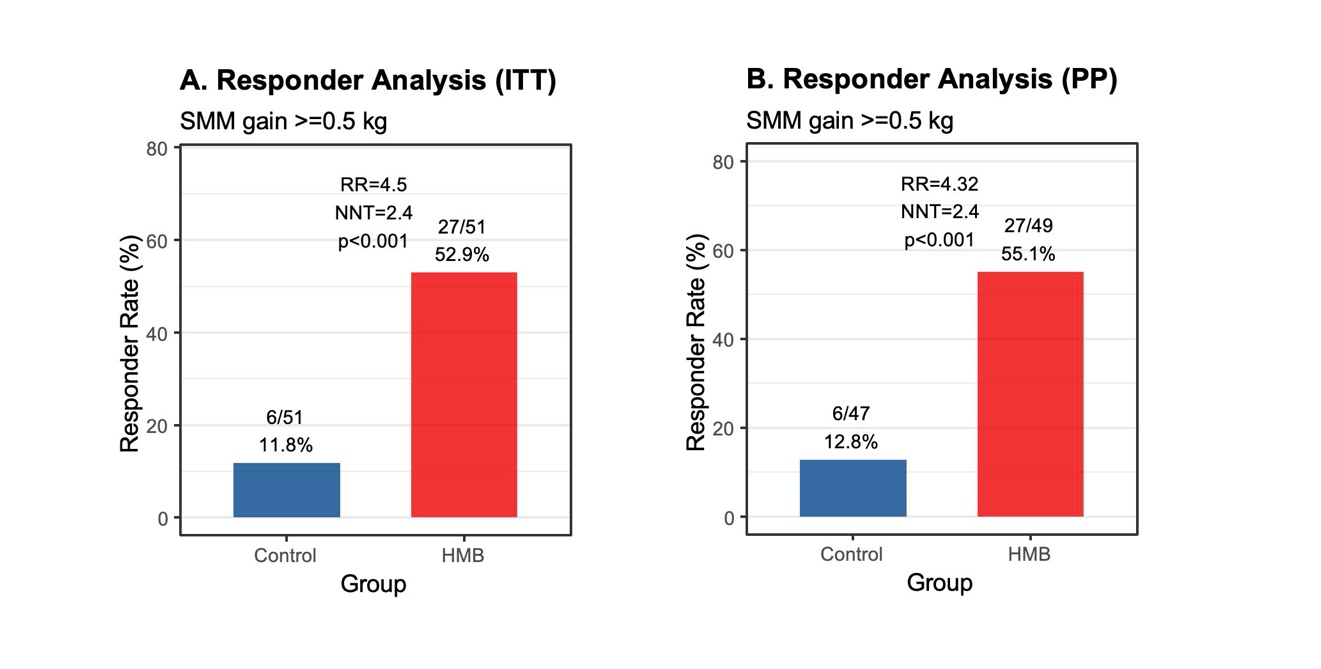
Figure S2

**Fig. S2 Effects of CaHMB-enriched supplementation on skeletal muscle mass during weight loss.** (A) Responder analysis showing the proportion of participants gaining ≥0.5 kg of SMM (ITT population); (B) Responder analysis showing the proportion of participants gaining ≥0.5 kg of SMM (PP population).

### Figure S3


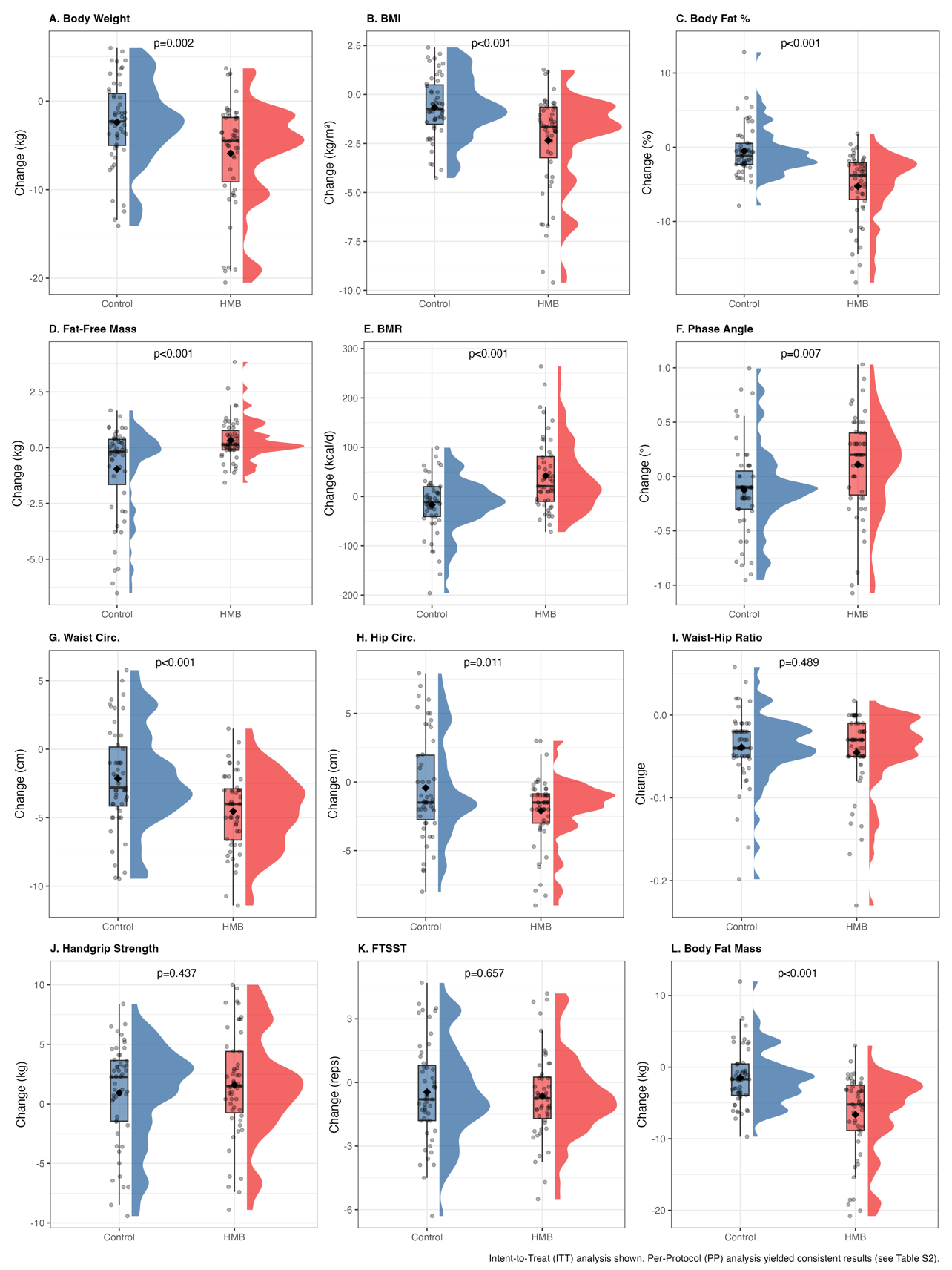
**Fig. S3: Detailed changes in body composition and physical function.**
Raincloud plots showing the distribution of changes from baseline to week 12 for all secondary body composition and physical function outcomes in the ITT population. (A) Body Weight, (B) BMI, (C) Body Fat Percentage, (D) Fat-Free Mass, (E) Basal Metabolic Rate, (F) Phase Angle, (G) Waist Circumference, (H) Hip Circumference, (I) Waist-Hip Ratio, (J) Handgrip Strength, (K) Five Times Sit-to-Stand Test (FTSST), (L) Body Fat Mass. P-values are from unadjusted group comparisons. Abbreviations: BMI, Body Mass Index; FTSST, Five Times Sit-to-Stand Test; ITT, Intention-to-Treat.

### Figure S4


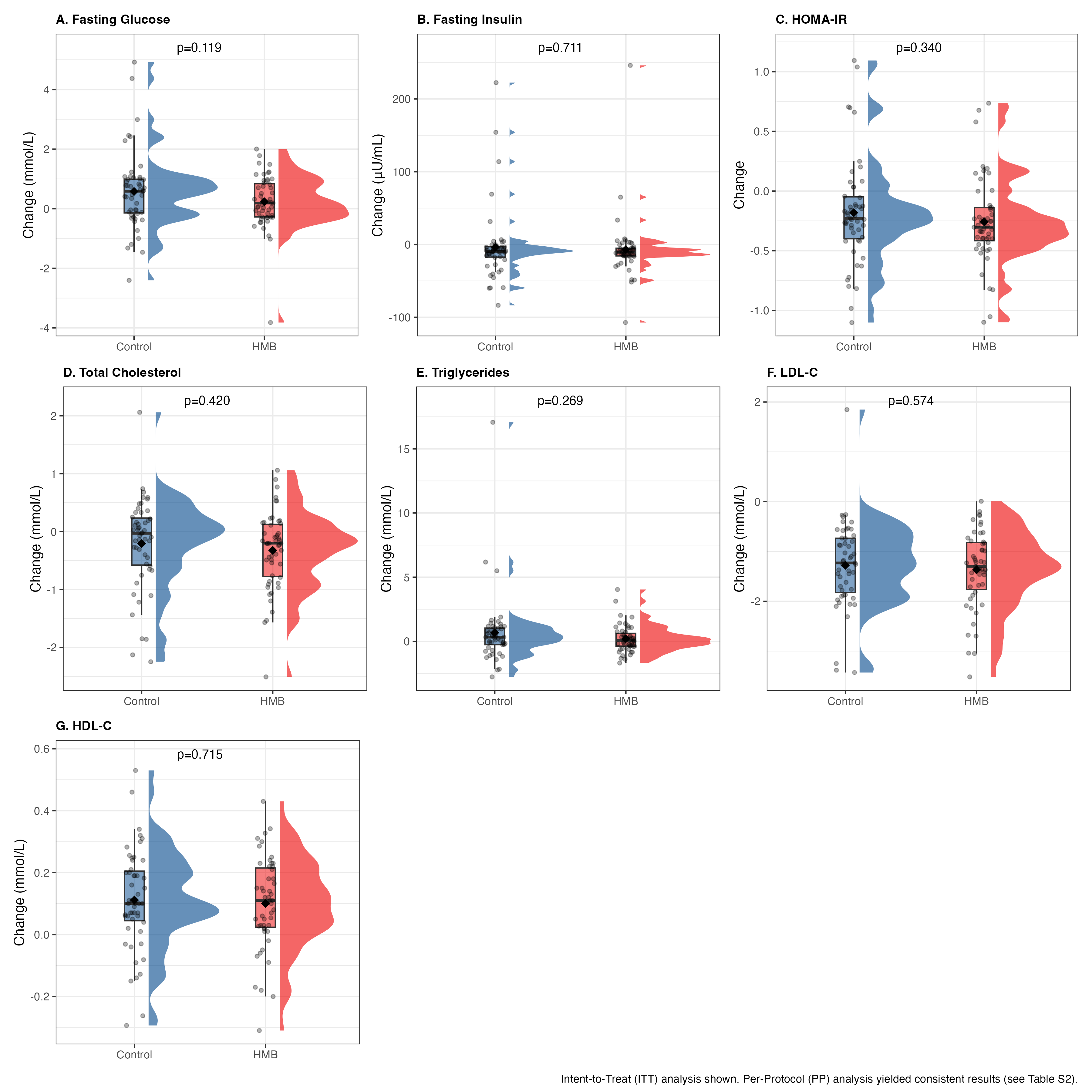


**Fig. S4: Detailed changes in metabolic biomarkers.**
Raincloud plots showing the distribution of changes from baseline to week 12 for metabolic outcomes in the ITT population. (A) Fasting Glucose, (B) Fasting Insulin, (C) HOMA-IR, (D) Total Cholesterol, (E) Triglycerides, (F) LDL-C, (G) HDL-C. Abbreviations: HOMA-IR, Homeostatic Model Assessment for Insulin Resistance; LDL-C, Low-Density Lipoprotein Cholesterol; HDL-C, High-Density Lipoprotein Cholesterol; ITT, Intention-to-Treat.

###
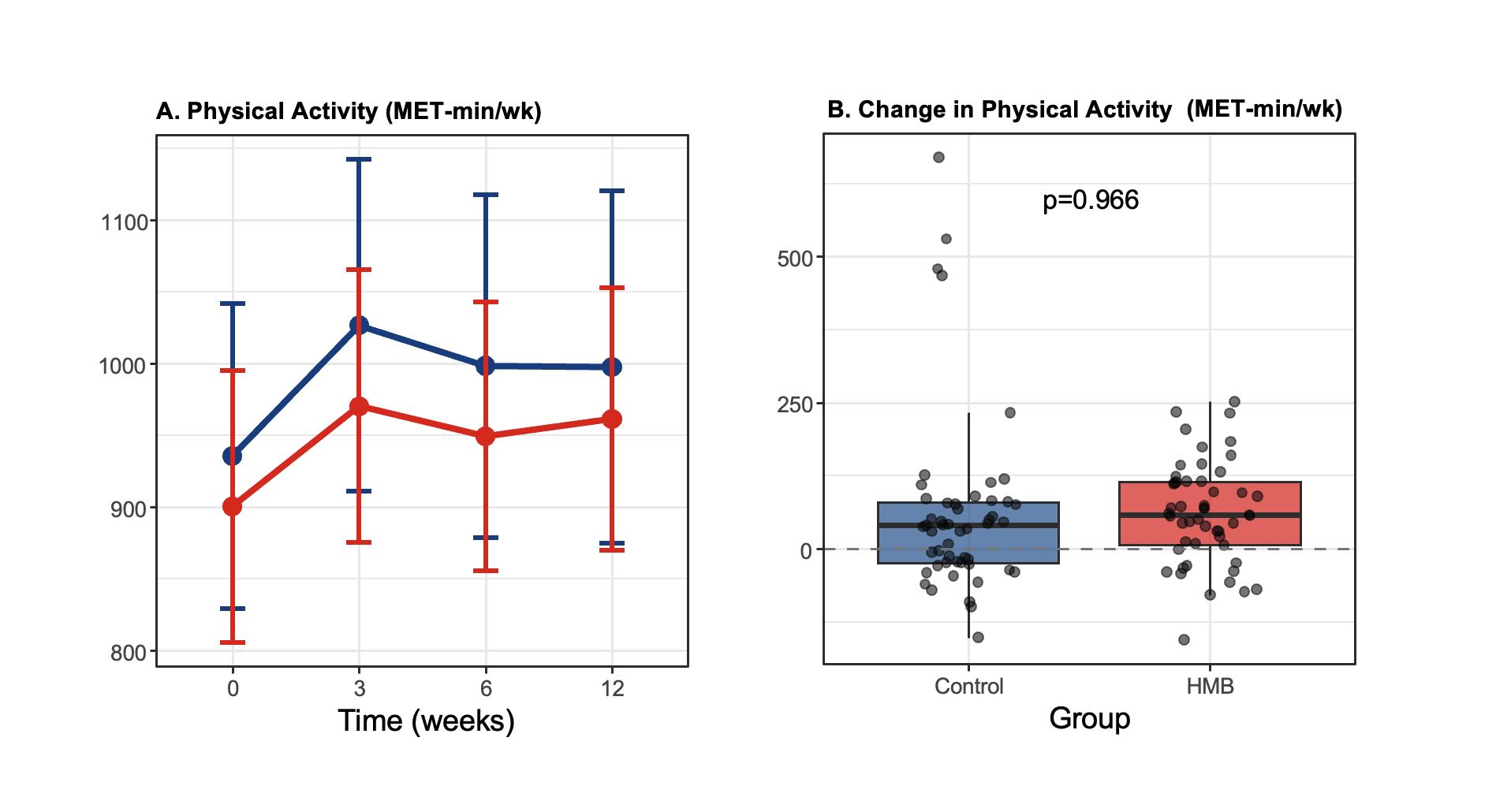
Figure S5

### Fig. S5: Physical activity trajectories. (A) Longitudinal trajectories of physical activity levels (IPAQ scores) throughout the 12-week intervention (mean ± SE); (B) changes in physical activity levels from baseline to week 12. Abbreviations: IPAQ, International Physical Activity Questionnaire; MET, metabolic equivalent of task; SE, standard error.

### Figure S6


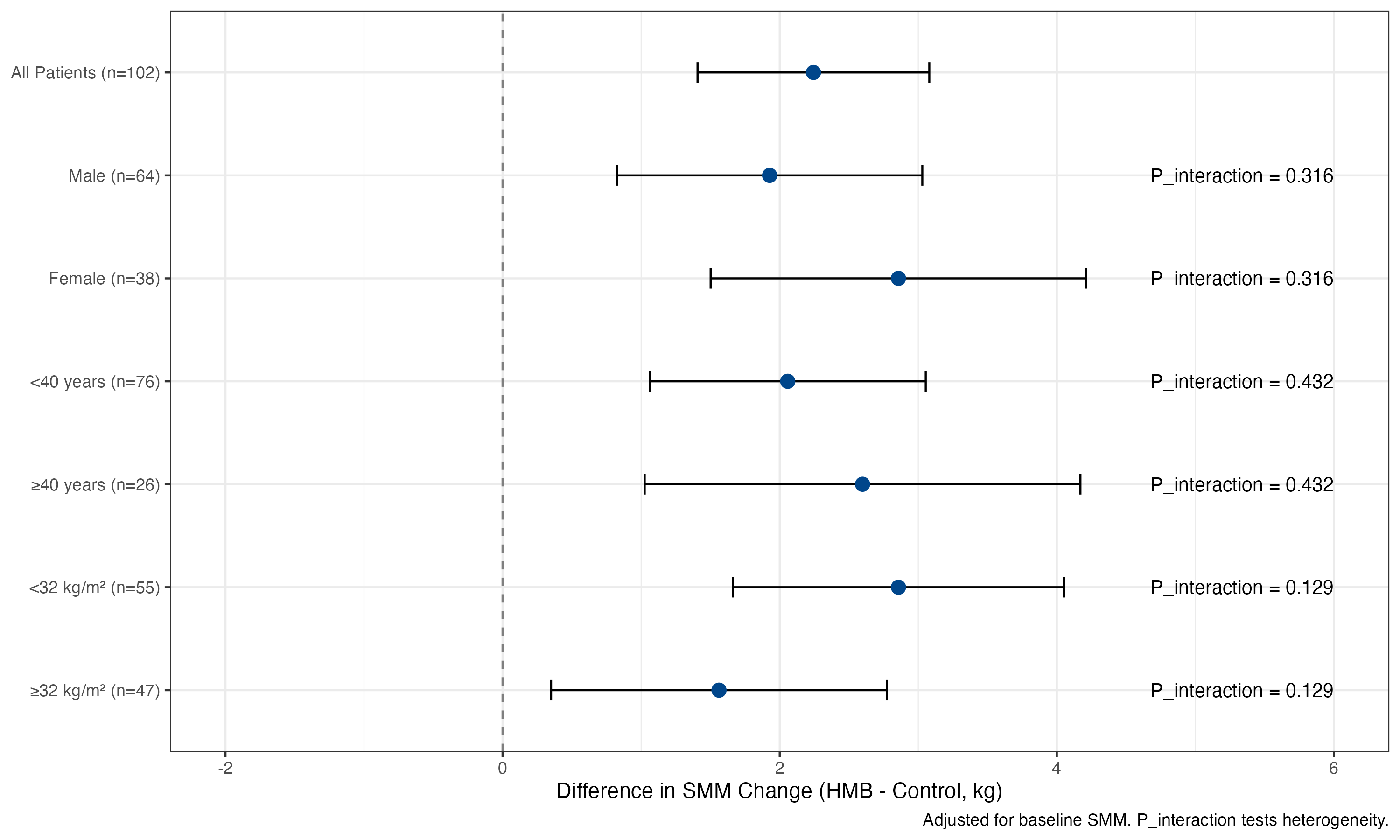


**Fig. S6: Subgroup analyses of the primary outcome.**
Forest plot displaying the treatment effect (difference in SMM change) across prespecified subgroups: Gender (Male vs. Female), Age (<40 vs. ≥40 years), and Baseline BMI (<32 vs. ≥32 kg/m²). P-values for interaction evaluate the heterogeneity of the treatment effect across subgroups. Analysis was performed on the ITT population, adjusting for baseline SMM. Abbreviations: SMM, Skeletal Muscle Mass; BMI, Body Mass Index; HMB, β-hydroxy-β-methylbutyrate.

### Figure S7


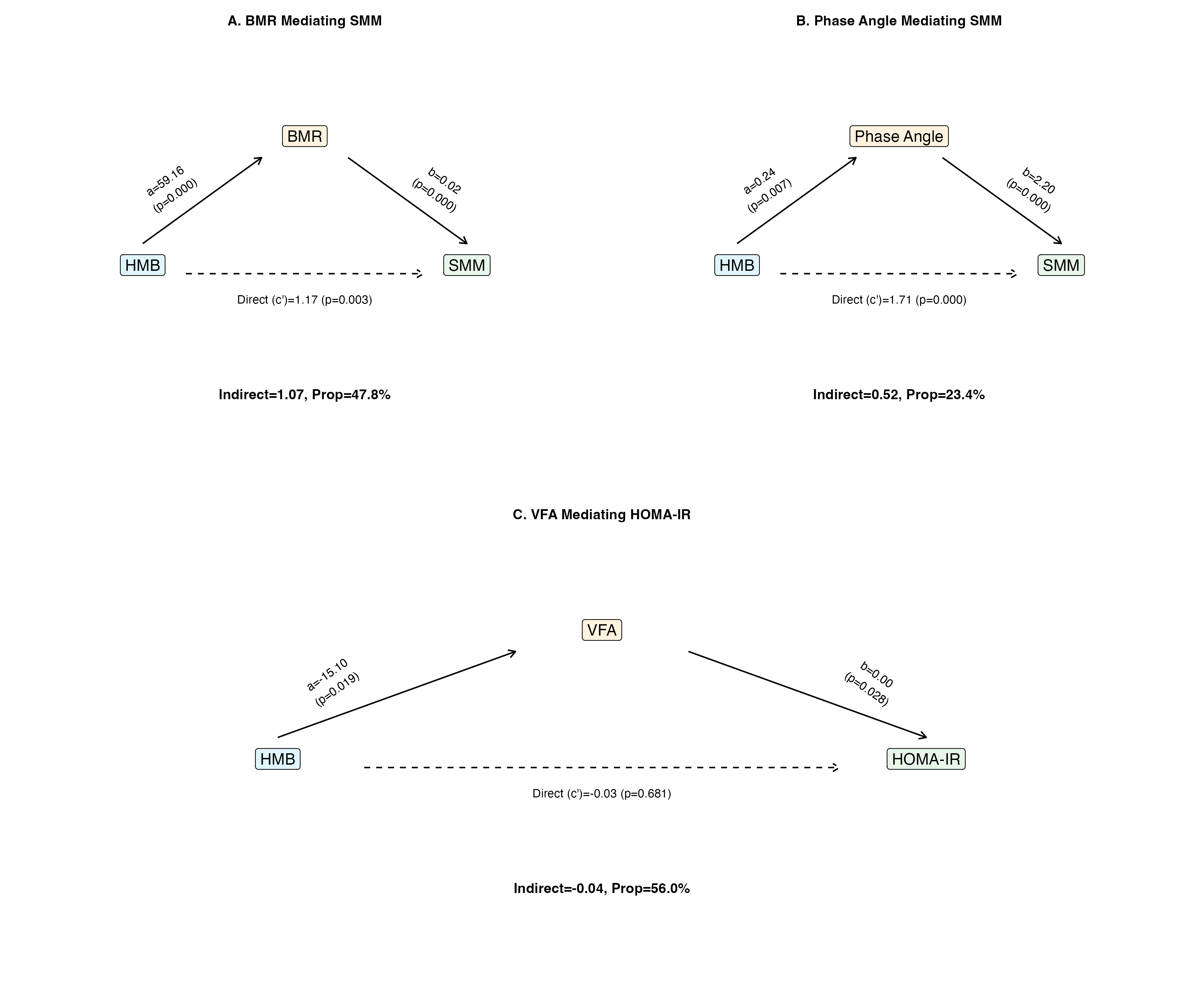


Fig. S7: Mediation Analysis of CaHMB Effects on Skeletal Muscle Mass.

Path diagrams illustrating the mediation models. (A) The effect of CaHMB-enriched supplementation on SMM mediated by changes in Basal Metabolic Rate (BMR). (B) The effect of CaHMB-enriched supplementation on SMM mediated by changes in Phase Angle. Coefficients (a, b, c’, and indirect effects) are presented with P-values. Abbreviations: SMM, Skeletal Muscle Mass; BMR, Basal Metabolic Rate.

### Figure S8


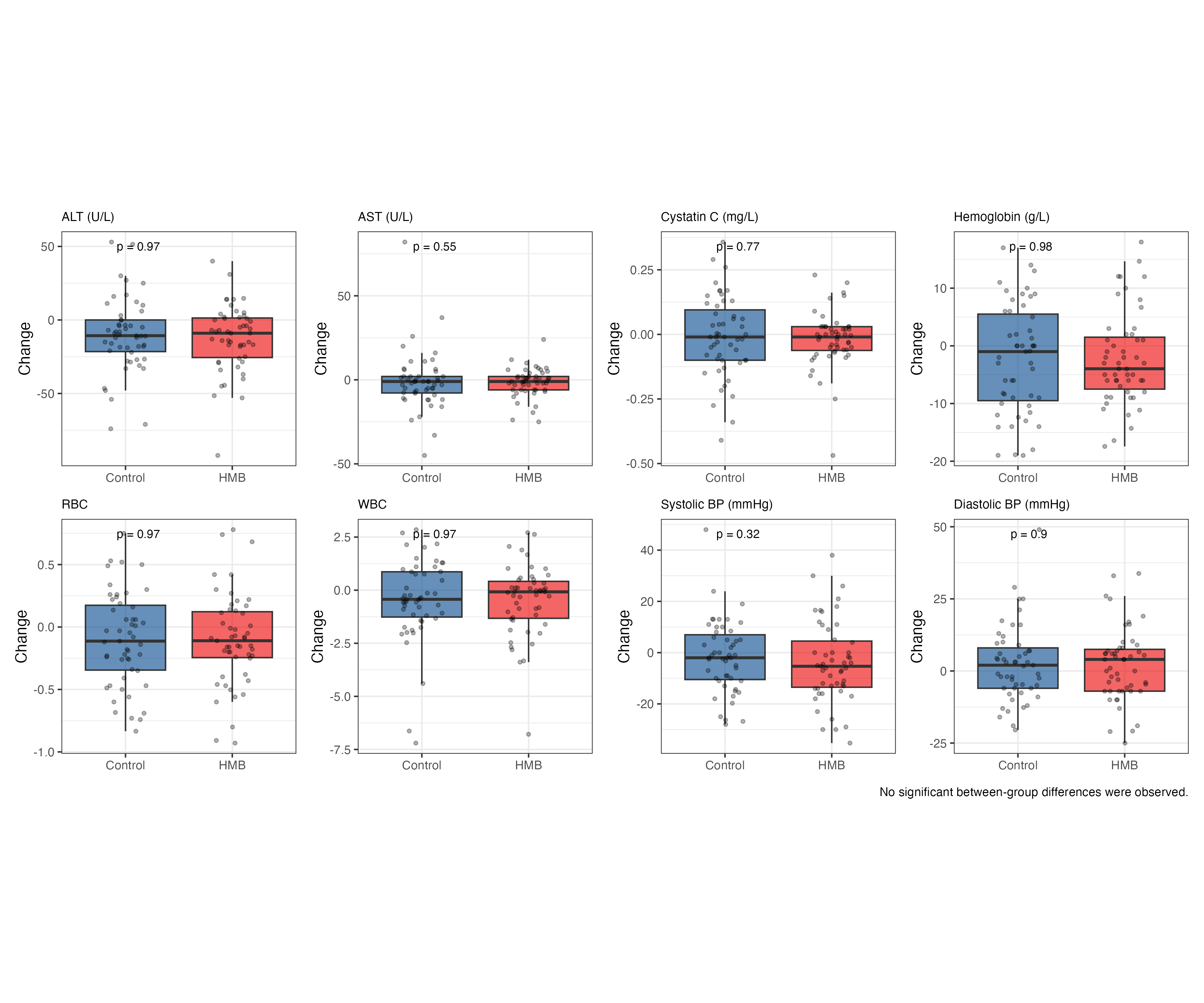


**Fig. S8: Safety evaluation.**
Boxplots comparing the changes in safety parameters from baseline to week 12 between groups. Parameters include liver function enzymes (ALT, AST, GGT), renal function markers (Creatinine, Cystatin C, eGFR), haematological indices (Hemoglobin, RBC, WBC), and blood pressure (SBP, DBP). No significant differences were observed. Abbreviations: ALT, Alanine Aminotransferase; AST, Aspartate Aminotransferase; GGT, Gamma-Glutamyl Transferase; eGFR, estimated Glomerular Filtration Rate; RBC, Red Blood Cell count; WBC, White Blood Cell count; SBP, Systolic Blood Pressure; DBP, Diastolic Blood Pressure.
